# Domains in Action: Understanding Ddi1’s Diverse Functions in the Ubiquitin-Proteasome System

**DOI:** 10.3390/ijms25074080

**Published:** 2024-04-06

**Authors:** Artur Fabijan, Bartosz Polis, Agnieszka Zawadzka-Fabijan, Izabela Korabiewska, Krzysztof Zakrzewski, Emilia Nowosławska, Michał Chojnacki

**Affiliations:** 1 Department of Neurosurgery, Polish-Mother’s Memorial Hospital Research Institute, 93-338 Lodz, Poland; jezza@post.pl (B.P.); krzysztof.zakrzewski@iczmp.edu.pl (K.Z.); emilia.nowoslawska@iczmp.edu.pl (E.N.); 2 Department of Rehabilitation Medicine, Faculty of Health Sciences, Medical University of Lodz, 90-419 Lodz, Poland; agnieszka.zawadzka@umed.lodz.pl; 3 Department of Rehabilitation, Faculty of Dental Medicine, Medical University of Warsaw, 02-091 Warsaw, Poland; iza.korabiewska@op.pl; 4 Department of Medical Biochemistry, Medical University of Lodz, 92-215 Lodz, Poland; chojnacki.michal84@gmail.com

**Keywords:** ubiquitin (Ub), DNA-damage inducible protein 1 (Ddi1), ubiquitin-proteasome system (UPS), ubiquitin-like domain (UBL), ubiquitin associated domain (UBA), retroviral-like protease domain (RVP)

## Abstract

The ubiquitin-proteasome system (UPS) is a pivotal cellular mechanism responsible for the selective degradation of proteins, playing an essential role in proteostasis, protein quality control, and regulating various cellular processes, with ubiquitin marking proteins for degradation through a complex, multi-stage process. The shuttle proteins family is a very unique group of proteins that plays an important role in the ubiquitin-proteasome system. Ddi1, Dsk2, and Rad23 are shuttle factors that bind ubiquitinated substrates and deliver them to the 26S proteasome. Besides mediating the delivery of ubiquitinated proteins, they are also involved in many other biological processes. Ddi1, the least-studied shuttle protein, exhibits unique physicochemical properties that allow it to play non-canonical functions in the cells. It regulates cell cycle progression and response to proteasome inhibition and defines MAT type of yeast cells. The Ddi1 contains UBL and UBA domains, which are crucial for binding to proteasome receptors and ubiquitin respectively, but also an additional domain called RVP. Additionally, much evidence has been provided to question whether Ddi1 is a classical shuttle protein. For many years, the true nature of this protein remained unclear. Here, we highlight the recent discoveries, which shed new light on the structure and biological functions of the Ddi1 protein.

## 1. Introduction

The ubiquitin-proteasome system is a non-lysosomal, ATP-dependent proteolytic system, selectively degrading cellular proteins. This system plays crucial role in maintaining proteostasis. Not only does it degrade proteins, it is also a crucial component of the protein quality control system, eliminating misfolded and aberrant proteins, which can affect the homeostasis of the cell. Due to its proteolytic character, UPS tightly controls and regulates many cellular key processes, e.g., cell signalling, cell cycle, immune response, mitochondrial dynamics, apoptosis, and DNA repair [1,2]. UPS-dependant degradation consists of two major processes including ubiquitination and degradation-marked proteins in the proteasome.

Ubiquitin is a small, highly conserved protein comprising 76 amino acids commonly found in eukaryotic organisms. It plays the role of a marker, pointing out proteins destined for degradation. Ubiquitination is a complex, multi-stage post-translational process. The entire process consists of three different types of reactions, where particular enzymes are involved: E1 (ubiquitin-activating enzyme), E2 (ubiquitin-conjugating), and E3 (ubiquitin ligase) and through cascading reactions they form polyUb chains. PolyUb chains are linked through isopeptide bonds involving the carboxyl terminus of one Ub and the ε-amine group of the lysine-side chain of another Ub or a target protein. Eight distinct Ub linkages are accounted for by linkage through Ub’s seven lysines (K6, K11, K27, K29, K33, K48, and K63) or the N-terminus methionine. Often, polyUb signals have been implicated in orthogonal processes; for example, K63-linked chains are involved in non-degradative pathways (e.g., DNA repair, oxidative stress, signaling and membrane trafficking) [3,4,5,6], whereas K48-linkages are the canonical signal for degradation of substrates at the proteasome [7]. Interestingly, the dogma that K48-linked polyUb is the only proteasomal targeting signal has been challenged, and it is apparent a diverse range of polyUb signals can be recognized by the proteasome [8]. The biological function of the atypical polyUb chains is still being thoroughly researched.

**Figure 1:**
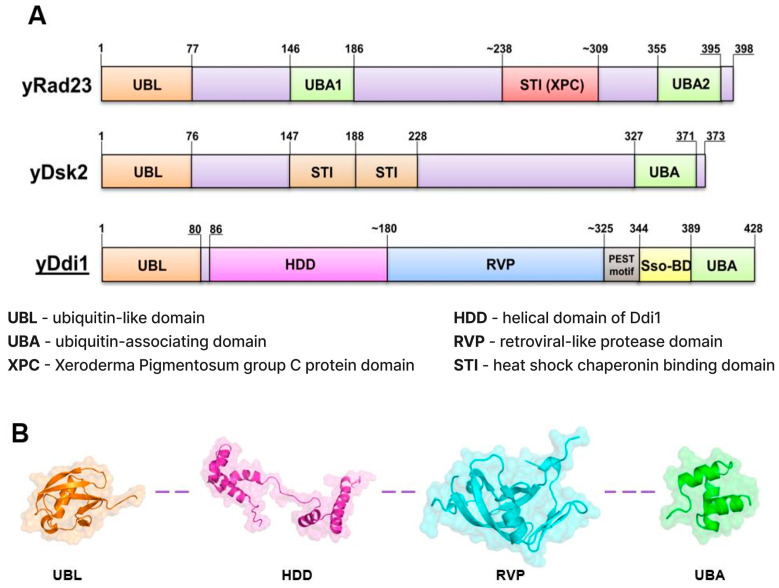
(**A**) Mapping of domains of yeast shuttle proteins (Rad23, Dsk2, Ddi1). (**B**) Cartoon representation of yDdi1’s solved structures of the domains: UBL (PDB:2N7E, orange), HDD (PDB:5KES, magenta), RVP (PDB:4Z2Z, cyan), and UBA (PDB:2MR9, green).

Shuttle proteins play a pivotal role in the UPS. These proteins possess both ubiquitin-like (UBL) and ubiquitin-associated (UBA) domains. The UBL domain enables them to bind to the proteasome, facilitating protein degradation, while the UBA domain allows them to interact with polyubiquitinated substrates. By bridging the proteasome and ubiquitinated proteins, shuttle proteins ensure the targeted degradation of proteins marked for destruction, maintaining cellular protein homeostasis [9]. How proteins destined for degradation are recognized by the proteasome and then degraded is poorly understood. However, polyubiquitin interaction with the proteasome is often mediated by several shuttle proteins (e.g., Dsk2/hPLIC/UBQLN, Rad23/ HR23A, and Ddi1/hDdi1/2). Proteasomal shuttle proteins are not associated with the 26S proteasome or its 19S regulatory particle. For these proteins to play the role of shuttles of ubiquitinated substrate, they have to comprise at least one ubiquitin-associated domain (UBA) and ubiquitin-like domain (UBL) [10,11,12]. The shuttles drive ubiquitinated proteins to the proteasome by binding the polyUb tag via the shuttle’s UBA domain. This domain has high affinity for ubiquitin and facilitates the selective recognition of ubiquitinated substrates within the cell. The UBL domain’s ability to mimic ubiquitin’s structural features allows it to interact with proteins containing ubiquitin-binding domains, facilitating substrate recognition and recruitment to the proteasome. This specific domain-based structure is essential for maintaining protein quality control and removing aberrant or damaged proteins from the cell, thereby preventing the accumulation of toxic aggregates and maintaining cellular homeostasis. Shuttle proteins exhibit considerable diversity, manifesting discernible distinctions among them, often characterized by the presence of additional domains tailored to augment their functions (Figure 1A).

All of these domains exhibit specific properties, which allows them to play divergent roles in cells. Delivery of ubiquitinated substrates to the proteasome requires the presence of specific receptors. So far, receptors Rpn1/S2/PSMD2, Rpn10/S5a, and Rpn13/Adrm1 were established as “docking stations” for polyUb conjugates [13,14,15,16,17]. They are located on the exposed surface of the 19S RP particle, bounded by their N-terminal regions to the proteasome. Receptors contain specific structural motifs which allow them to bind ubiquitin or UBL domains of shuttle proteins. Rpn10 binds ubiquitin via the C-terminal UIM domain, Rpn13 binds ubiquitin via the pleckstrin-like Pru domain, and Rpn1 via PC repeats region—T1. Rad23 and Dsk2 are able to bind to Rpn10 and Rpn13 and Rpn1, whereas for Ddi1, the only identified receptor is Rpn1 [18]. However interaction between Ddi1 and Rpn1 is the weakest among all the shuttle proteins [19].

The Ddi1, the least studied member of the UBL-UBA family proteins, functions as a shuttle protein, and facilitates the degradation in the ubiquitin-proteasome system. The Ddi1 protein was firstly described as transcriptionally induced leading to subsequent overexpression in response to genotoxic factors leading to DNA damage [20]. Notably, Ddi1 displays heightened sensitivity to any impairment or inhibition of the proteasome [21,22]. Its structural composition includes the N-terminal UBL domain (1–80), the RVP domain (180–350), and C terminal UBA domain (389–428) (Figure 1B). The RVP domain is distinctive to Ddi1, setting it apart from other shuttle proteins, contributing to its distinct functional properties and cellular roles. Despite sharing a similar structure to other shuttle proteins, Ddi1 domains possess unique properties, rendering the protein a compelling subject for further research.

## 2. Ddi1, Rad23, and Dsk2: Shared Characteristics and Contrasts

Ddi1, Rad23, and Dsk2 are all shuttle proteins involved in ubiquitin-mediated protein degradation pathways, yet they exhibit notable differences in their structural and functional characteristics. Yeast Rad23 and its human homolog hHR23A contain an N-terminal ubiquitin-like (UBL) domain and two ubiquitin-associated (UBA) domains, which bind ubiquitin chains (primarily of the K48 type) composed of at least four units [23]. Human Rad23A and Rad23B are expressed in most human tissues, with RAD23B being more abundant. Homologs in different species predominantly localize in the nucleus and, to a lesser extent, the cytosol. The NMR structure of the Rad23UBA2-K48-Ub2 complex revealed that UBA2 interacts simultaneously with both subunits of the dimer [24] Ub units binding to UBA2 through their canonical, exposed hydrophobic surface (L8, I44, V70). The UBL domain exhibits strong affinity for 19S proteasome receptors, particularly Rpn10 [25]. Beyond its role as a shuttle factor for conveying ubiquitinated substrates to the proteasome, it also contributes to additional cellular functions and pathways. Rad23 is mainly localized in the cell nucleus, where it plays a crucial role in NER (Nucleotide Excision Repair), especially those induced by UV light [26]. The XPC-binding (XPC) domain enables Rad23 to bind to XPC, a key protein in NER [27,28]. In the context of NER, Rad23’s partnership with XPC is vital for recruiting XPC to sites of DNA damage. Rad23 is believed to transport XPC to the damage site and dissociate before the final complex formation [29,30]. It also plays a significant role in stabilizing XPC, thereby enhancing its activity [31]. Additionally, Rad23 is involved in the ERAD pathway, aiding in the degradation of misfolded proteins from the endoplasmic reticulum. This involves interactions with components like PNGase, highlighting its involvement in various degradation pathways [32,33]. Similar to Rad23, Dsk2 binds to the K48-linked polyubiquitin (consisting of at least Ub_4+_) chains through its UBA domain [34]. The structure of the Dsk2UBA-Ub complex exhibits analogous characteristics to those observed in Rad23. The interaction has a classical hydrophobic character. Although the main proteasomal receptor for Dsk2 is Rpn10, the interaction is weaker than that of Rad23-Rpn10 [14]. Dsk2UBL can also bind to other 19S proteasome receptors but with significantly lower affinity [19]. Both Rad23 and Dsk2 cooperate in the degradation of ubiquitinated substrates, although it is suggested that under pathological conditions, they may protect them from degradation [35]. Rad23 and Dsk2 partially overlap in function, as deletion of one of them does not lead to drastic changes in the cell. However, double mutants ΔDsk2 and ΔRad23 exhibit significant disruptions in cell function [36]. Conversely, the level of Dsk2 must be tightly controlled, as its overexpression leads to cell death.

## 3. Ddi1’s Atypical UBL Domain

**Figure 2:**
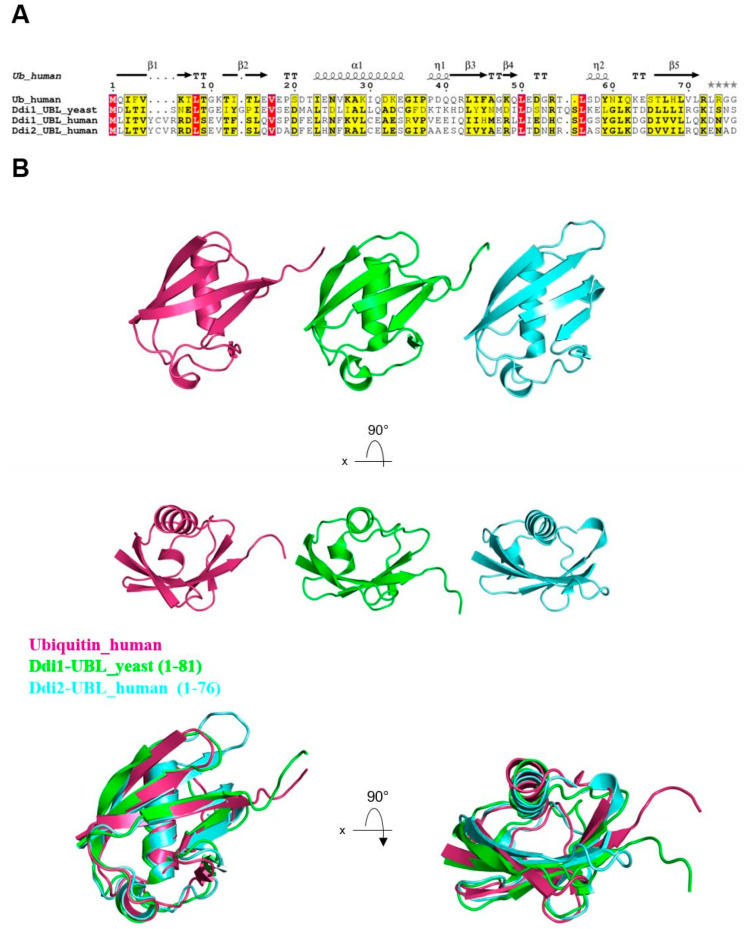
(**A**) Sequence alignment of ubiquitin and UBL domains of yeast Ddi1, human Ddi1, and human Ddi2. Kalign alignment software (version 2.0) was used [38] and output files were modified in ESPript software (version 3.1) [39]. Red indicates all residues are identical. Yellow-highlighted residues are similar in the group (in-group scores > 0.7). (**B**) Structures of Ub (PDB: 1UBQ, magenta), UBL of yeast Ddi1 (PDB:2N7E, green), and UBL of human Ddi2 protein (PDB:2N7D, cyan) and alignment of Ub molecule and UBL domains.

The UBL domain of shuttle proteins plays a crucial role in their ability to directly interact with the proteasome receptors, specifically Rpn1—a subunit of the 19S proteasome regulatory particle [18,37]. The UBL domain of yeast Ddi1, while sharing only a 14% sequence similarity with human ubiquitin (Figure 2A), exhibits high structural similarity to other UBLs within the shuttle protein family (Figure 2B). Structural resemblance facilitates interactions with ubiquitin-binding proteins and proteasomal components.

Its primary function is to deliver the ubiquitinated substrates to the proteasome and facilitate interaction with Rpn1. Rpn1’s structural features, including nine conserved leucine-rich regions (LRRs), enable its interaction with various proteins, and its position on the proteasome facilitates its role as a docking site for proteasome-associating factors. Ddi1-UBL directly binds to Rpn1, and a single amino acid mutation in Rpn1 (D517A) significantly reduced this interaction [18]. The UBL domain of Ddi1 exhibits a remarkable capacity for direct interaction with ubiquitin, a feature not commonly observed among UBL proteins. Structural analyses reveal key residues within the UBL domain that mediate this interaction with ubiquitin. The hydrophobic patch of Ddi1-UBL is surrounded by negatively charged aspartic acid (Asp) residues in the β-sheet, a unique feature compared to other UBLs. This hydrophobic patch, consisting of Leu-27, Leu-40, and Leu-41 in the α-helix, and Leu-71 and Ile-73 in the β5 strand, serves as the binding site for Rpn1. Recent studies have demonstrated a distinct charge distribution in Ddi1-UBL compared to Rad23-, Dsk2-UBLs, and ubiquitin [40]. In neutral pH, the β-sheet of Ddi1-UBL is negatively charged, while the β-sheet surface of Rad23 is positively charged. This charge reversal between Ddi1-UBL and ubiquitin promotes their interaction, involving residues Ile-13, Leu70, and Lue-72 in the Ddi1-UBL α-helix and β5 strand and the standard hydrophobic surface of ubiquitin (Leu-8, Ile-44, Val-70) [40]. The biological significance of this interaction remains to be elucidated. In contrast, the interaction between mammalian isoform of the Ddi1 homolog 2 and ubiquitin is much weaker, and no interaction was observed with ubiquitin dimers [41]. The ability to bind ubiquitin suggest that the Ddi1′s UBL domain may not conform to the characteristics of a canonical ubiquitin-like domain [19]. Additionally, the UBL domain of Ddi1 has been shown to form heterodimers with Rad23, specifically with the internal UBA1 domain of Rad23. This interaction requires a twofold molar excess of Rad23, and it is completely abolished in the Rad23-ΔUBA1 strain. The biological implications of this heterodimerization remain unclear [42,43].

## 4. Ubiquitin Recognition: Yeast UBA Domains and Human UIM Motifs

Yeast Ddi1 recognizes and binds to ubiquitinated proteins through its UBA domain. The canonical UBA domain (arranged by three α-helices) adopts a compact, globular form, which enables the UBA domain to form a stable complex with ubiquitin. The UBA domain enables Ddi1 to interact specifically with ubiquitin moieties attached to the target protein, thereby facilitating their delivery to the proteasome for degradation. The UBA domain of the Ddi1 serves as a versatile module that can recognize various types of ubiquitin chains, e.g., K48 and K63 [40,42]. The *Schizosaccharomyces pombe* ortholog of Ddi1, Mud1, binds K48-Ub2 with increasing affinity as the ubiquitin chain lengthens [44]. This suggests that the UBA domain may play a protective role by preventing the ubiquitination of Ddi1, which would otherwise make it susceptible to degradation by the proteasome [45]. The UBA domain, like the UBL, is also capable of forming heterodimers. At equimolar protein concentration, the Rad23′s UBL domain interacts with the Ddi1′s UBA domain [46]. In yeast, Ddi1 harbors a UBA domain at its C-terminus, facilitating the recognition and binding of ubiquitinated substrates. In contrast, Ddi1 human homologs lack the UBA domain and possess a short UIM motif (376–396) facilitating binding with monoubiquitin. No strong interactions with polyUb or Nedd8 were detected.

## 5. Combined Role of UBL and UBA

The Ddi1 serves as a pivotal regulator of the SCF^Ufo1^, a crucial E3 ligase that promotes protein breakdown via ubiquitination. The SCF^Ufo1^ comprises cdc53/cullin (yeast/human), SKP-1 and F-box proteins. The SCF^Ufo1^ primarily targets G1 cyclins essential for CDK kinase regulation, crucial in controlling cell cycle progression and DNA synthesis [47]. Within this complex, F-box protein Ufo-1, whose expression is induced by DNA damage, aids in substrate recruitment and ubiquitination. Notably one such substrate is the Ho endonuclease, which regulates yeast MAT-type by inducing double-strand DNA breaks during G1 phase. The Ho endonuclease is responsible for changing the MAT type of yeast. Ubiquitination of Ho endonuclease by the SCF^Ufo1^is mediated by Ddi1, playing a crucial mediating role in determining MAT type [48]. The combined action of the Ddi1’s UBL and UBA domains is integral; the UBA domain facilitates delivery of phosphorylated Ho endonuclease to the SCF^Ufo1^, while the UBL domain binds to the C-terminal tUIMs of Ufo-1 [49,50,51]. The knockout mutant ΔDdi1 causes the stabilization and accumulation of the Ho endonuclease in the cytoplasm, despite the presence of the other shuttle proteins like Dsk2 and the Rad23. The deletion of the tUIM motifs in F-box protein Ufo-1 abolishes the interaction with the Ddi1. However, the isolated tUIM alone are capable of binding to all of the shuttle proteins. The deletion of the UBL domain nullifies that interaction, whereas the deletion of the UBA domain has a lesser effect. The interaction between the Ufo-1 and the Ddi1 is crucial for Ufo-1 degradation and SCF^Ufo1^ turnover. Additionally, a specific point mutation identified in the Rpn1 subunit of the proteasome (D517A) results in the stabilization of substrates targeted by Ddi1. This observation suggests that Rpn1 functions as a docking site for Ddi1 within the proteasome complex [18].

Ddi1 is proposed to function as an adaptor protein, mediating the recruitment of chaperone Cdc48/p97/VCP to distinct cellular locales and substrates. This cooperative interaction is indispensable for the extraction of misfolded proteins; Ddi1 likely targets misfolded proteins, such as Cps1 (carboxypeptidase S), and enlists Cdc48 to extract them from cellular structures, thereby averting aggregation and potential cellular damage. Moreover, Ddi1 is implicated in the anterograde transport of select proteins to the yeast vacuole. Its association with Cps1 and involvement in the constitutive anterograde transport to the multivesicular body and vacuolar lumen underscore the broad spectrum of cellular functions attributed to Ddi1, despite its primary role not being directly associated with DNA integrity maintenance. The direct interaction between Ddi1 and Cdc48 obviates the necessity for any additional factors such as Npl4 or Ufd1. The UBA and UBL domains of Ddi1 play a pivotal role in facilitating this interaction [52].

## 6. SSO-BD Domain

**Figure 3:**
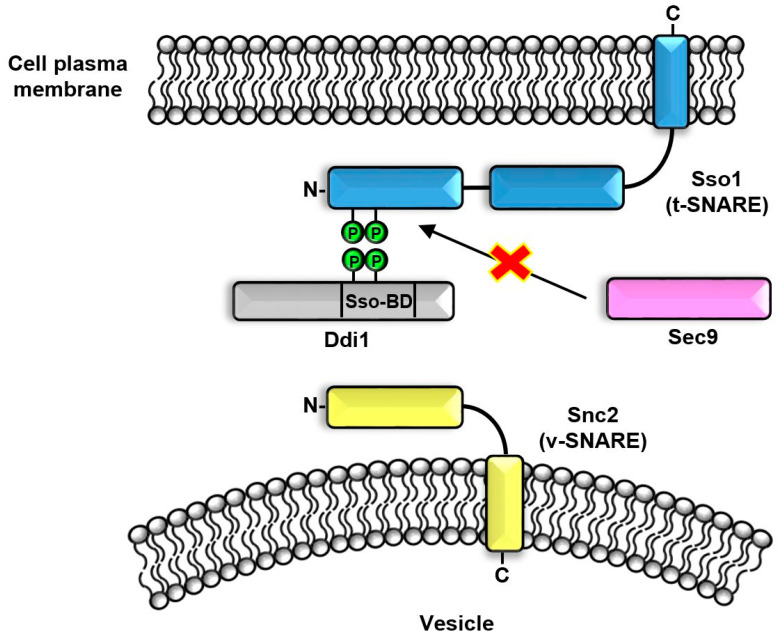
Divergent non-canonical cellular roles of the Ddi1. The Ddi1 blocks interaction between Sso-1 (t-SNARE) and Sec9.

The Ddi1 was identified as a SNARE (Soluble NSF Attachment Receptor)-interacting protein which directly interacts with v-SNARE (Snc1, preferably Snc2) [53] and t-SNARE (Sso1) proteins [54], thereby regulating exocytosis by negatively impacting protein secretion. Ddi1 binds to the N-terminal domain of the t-SNARE protein Sso1, preventing its interaction with its functional partner Sec9 (Figure 3). Ddi1 acts as a competitive inhibitor for Sec9, ultimately leading to exocytosis inhibition. The interaction between Ddi1 and Sso1 is facilitated by phosphorylation of the N-terminal domain of the Sso1 and the Ddi1 region between the RVP and the UBA domains’ (called Sso1 Binding Domain, 344-395aa) residues T348 and T346. The PEST motif located between the RVP and UBA domains may further enhance this interaction [21]. Additionally, Ddi1 interacts with other t-SNARE proteins Tlg1 and Tlg2, further contributing to its inhibitory effect on protein secretion. The binding of Ddi1 to Snc2 prevents Snc2 from forming the SNARE complex, which is a critical step in the fusion process that delivers secretory vesicles to the cell membrane. This disruption of the SNARE complex ultimately leads to the inhibition of exocytosis. In summary, overexpression of the Ddi1 protein diminishes overall exocytosis by preventing interactions between v- and t-SNARE proteins.

## 7. Ddi1’s Unique RVP Domain

The Ddi1 protein uniquely contains the RVP domain, which is conserved among all Ddi1-related proteins [55]. The RVP domain has a low sequence similarity to the retroviral protease; nevertheless it contains a catalytic triad motif (*Saccharomyces cerevisiae*-DTGA, *H. sapiens*-DSGA). The RVP’s crystal structure demonstrates a remarkable similarity to aspartic protease family folding [41,56]. It is sensitive to HIV anti-retroviral drugs (proteinase inhibitors) in *Leishmania* [57]. Additionally, it has been shown to be a very important and crucial feature of the RVP domain of Ddi1. It reveals a strong tendency to form dimers via direct interaction. Creating dimers only via the RVP domain leaves the UBL and UBA domains unbound.

Shuttle proteins play a critical role in regulating the cell cycle, specifically the transition from the G2 phase to the M phase and the separation of sister chromatids during anaphase [58,59]. The presence of all domains including UBL, RVP, and the UBA is important to regulate the Pds1 stability. Active Pds1 binds to and inhibits Esp1, a cysteine protease that cleaves proteins involved in sister chromatid separation [60]. In response to DNA damage, shuttle proteins are overexpressed, potentially leading to cell cycle arrest. Ddi1 binds ubiquitinated Pds1, preventing the elongation of the ubiquitin chain that is assembled by the APC complex [59]. This action protects Pds1 from degradation by the proteasome. Whether Ddi1 directly interacts with the APC complex remains unclear, but it is likely that Ddi1 inhibits chain elongation, thereby safeguarding Pds1 from proteasome degradation.

**Figure 4:**
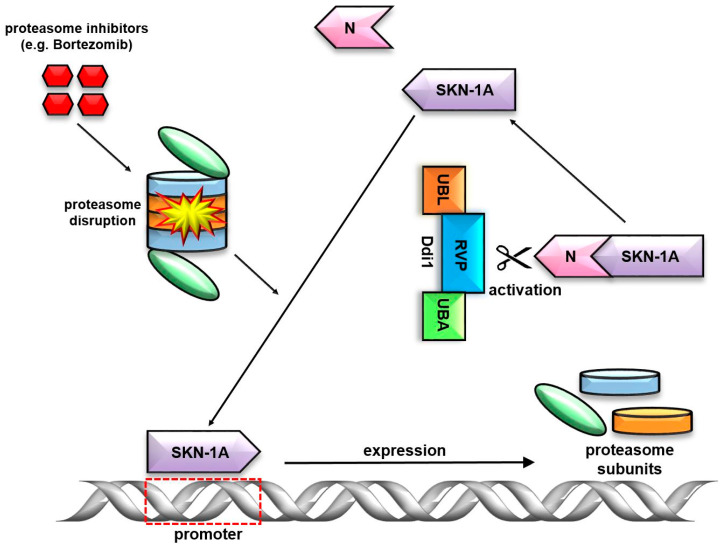
Divergent non-canonical cellular roles of the Ddi1. Positive feedback regulation of the SKN-1A. Followed by the proteasome disruption the SKN-1A as a transcription factor induces the expression of the Ddi1. The RVP domain of the Ddi1 activates the SKN-1A by cleaving the N-terminal polypeptide.

For many years, the enzymatic role of the RVP was unknown. Several studies attempted to identify its DUB activity, but these efforts were unsuccessful. The research conducted by Sha and Goldberg provided the first significant insights. It demonstrated the essential role of the transcription factor Skn-1 (*C. elegans*) and confirmed in Nrf1 (*H. sapiens*) in proteasome rescue [61,62]. When proteasome malfunctions occur, the Ddi1 and Skn-1A undergo changes in their expression levels. Surprisingly, Ddi1 was identified as the activator of Skn-1A, an isoform of Skn-1. To activate the ER-localized transcription factor Skn-1A, its N-terminal polypeptide requires deglycosylation followed by cleavage by Ddi1-RVP. The activated form of Skn-1A translocates to the nucleus and induces the expression of target genes, including proteasome subunits (Figure 4). Moreover, these results demonstrated that not only the RVP domain but also the UBL domain are essential for this process [63]. Human Ddi1 homolog 2 (Ddi2) functions in a similar manner [64]. The nature of the interaction, whether it entails direct engagement or involves a multi-stage process, where Ddi1 serves as a cofactor, remains elusive [22,65]. These findings have the potential to significantly advance our understanding of bortezomib resistance development and enhance the efficacy of chemotherapy.

Similar to the proteasome, Ddi1 possesses the capability to degrade ubiquitinated proteins. It exhibits strict specificity for K48-linked polyubiquitin chains of a certain length The exact cleavage site in the substrate is not determined, but it appears to be similar to that in Nrf1. The recognition motif for Ddi1 is located near the lysine residue where the polyubiquitin chain is attached to the target protein. The minimal ubiquitin-binding unit of Ddi1 comprises the HDD and RVP domains, while the UBL or UBA domains contribute to enhanced substrate affinity and proteolytic activity. These structural and functional features distinguish Ddi1 from the proteasome and suggest a distinct mechanism for polyubiquitinated substrate degradation [66].

Ddi1 possesses a high affinity for polyubiquitinated substrates. It acts as a complement to the proteasome but is not essential for cell viability under normal conditions. Enzymatically inactive Ddi1 mutants lead to the accumulation of polyubiquitinated proteins, indicating the existence of Ddi1 substrates even in untreated cells. Ddi1 may serve as a safeguard to cleave undegraded polyubiquitinated proteins into smaller peptides when proteasome function is compromised. Ddi1 and Ddi2 are unique in their ability to cleave proteins with long ubiquitin chains. This raises the possibility of using Ddi2 inhibition as a therapeutic strategy in cancer therapy, possibly in combination with proteasome inhibition [64,66].

At the molecular level, human homologs Ddi1 and Ddi2 regulate the Replication Termination Factor 2 (RTF2) at the stalled replisome, crucial for replication fork stabilization and restart. This regulatory mechanism is essential for the accurate and timely transmission of genetic information, highlighting Ddi1’s role in ensuring genome stability and its necessity for normal replication fork speeds during DNA replication. Its involvement in the degradation of the replication termination factor RTF2, facilitating the restart of stalled replication forks, underscores its multifaceted role in ensuring genomic integrity [67,68,69]. Further research on yeast Ddi1 have demonstrated that RVP (catalytic aspartate, Asp-220) and HDD (148–191aa) domains are crucial in countering DNA replication stress induced by hydroxyurea. Notably, the combined deletion of Ddi1 and Wss1 (weak suppressor of Smt3) in yeast leads to increased sensitivity to hydroxyurea, underlining the importance of Ddi1 in the cellular mechanisms for coping with DNA damage. This is further supported by Ddi1’s role in a genome-wide synthetic lethality screen with Wss1, showing a strong negative genetic interaction that emphasizes its significance in replication stress management [70].

The DNA-protein crosslink (DPC) poses a significant challenge to genomic integrity and cellular function, as it impedes DNA replication, transcription, and repair processes. Ddi1 and Wss1 emerge as crucial players in the resolution and repair of DPCs, each contributing distinct yet complementary roles in safeguarding genome stability. Ddi1’s involvement in DPC repair, particularly its recruitment to persistent DPC lesions and contribution to the efficient disassembly of these complexes, further underscores its critical role in maintaining cellular integrity. In the absence of Wss1, Dsi1 exhibits functional compensation by assuming its role, thereby conferring protection against DPCs. Impairment of the catalytic activity by introducing point mutation in the RVP domain (D220A) leads to accumulation of Rpb1 (subunit of the RNA polymerase II) on chromatin. Moreover, Ddi1 is recruited to persistent DPC lesions, such as stabilized topoisomerase-1 cleavage complexes (Top1ccs), in an S phase-dependent manner to assist in the eviction of crosslinked proteins from DNA [71].

Ubx5, an adaptor protein of Cdc48, accumulates at persistent DPC lesions in the absence of Wss1, hindering their efficient removal from DNA. Disruption of Ubx5-CDC48 binding or complete loss of Ubx5 reduces the sensitivity of wss1Δ cells to DPC-inducing agents, favoring the utilization of alternative repair pathways. Ddi1 contributes to the degradation of stalled RNA polymerase II (RNAPII) in the absence of Wss1 and Ubx5. Overall, Ddi1 emerges as a significant participant in the proteolysis of DNA-bound proteins and the repair of DPCs, operating in concert with other proteins such as Wss1, Cdc48, Ubx5, and potentially Ubx4. Further research is imperative for a comprehensive understanding of Ddi1’s specific roles and interactions in DPC repair [72].

Protease activity which is provided by the RVP domain plays a role in regulating proteasome condensation formation. Unlike Rad23 and Dsk2, Ddi1 does not show a strong direct dependence in proteasome condensation formation. However, experiments showed that Ddi1 (D220N), a protease catalytically inactive mutant, showed accumulation of long K48-polyubiquitin chains. It is hypothesized that these polyUb chains may act as a scaffold facilitating the formation of the proteasome condensation. An active RVP domain indirectly limits the formation of condensates by interacting with long K48-polyubiquitn chains [73].

## 8. The Helical Domain Ddi1 (HDD)

The Helical Domain Ddi1 (HDD) in yeast’s Ddi1 protein constitutes a pivotal structural and functional element, exhibiting intricate design and potential biological significance. Structurally, the HDD is composed of two alpha-helical domains: the N-terminal domain (residues 89–141) forms a four-helix bundle with a hydrophobic core (displaying high similarity with the DNA interacting domains), and the C-terminal domain (residues 150–190) consists of a two-helix hairpin with a smaller hydrophobic core. These domains are interconnected by a 10-residue linker, characterized by lower heteronuclear NOE values and minimal sequence conservation among Ddi1 orthologs, enabling dynamic interdomain interactions without direct packing [51].

Functionally, the HDD may play a crucial role in protein-protein interactions, potentially facilitating the Ddi1 protease’s interaction with substrates. It shares homology with Sti1-like domains involved in protein-protein interactions. Furthermore, the HDD demonstrates structural similarities to DNA-binding domains in transcriptional regulators, suggesting a potential role in DNA motif recognition. This is further supported by the presence of a conserved basic residue (Arg-131), which aligns with the DNA phosphate backbone in structural alignments with DNA-binding proteins. This alignment suggests a possible involvement of the HDD in DNA binding, particularly at DNA damage sites requiring ubiquitin-dependent proteolysis, aligning with Ddi1’s role in the DNA damage response and cell cycle checkpoints [51].

## 9. Future Perspectives

The Ddi1 protein and its associated domains present a rich tapestry of research opportunities. A profound exploration of the RVP domain is warranted, particularly focusing on its enzymatic functionalities, its potential DUB activity, and its established role in catalysing the Skn-1A transcription factor. Concurrently, the interactions of the UBL and UBA domains with other proteins merit scrutiny, especially their contributions to the ubiquitin-proteasome system and their speculated involvement in DNA repair mechanisms.

The HDD domain is a crucial domain to retain proteolytic activity of Ddi1, which beckons a comprehensive elucidation. Similarly, the implications of the SSO-BD domain’s interactions with SNARE proteins and its regulatory role in exocytosis demand a deeper understanding.

From a therapeutic standpoint, the potential applications of Ddi1 and Ddi2 in oncology, especially when paired with proteasome inhibition, offer promising avenues. This is complemented by the need to pinpoint the exact cleavage sites and recognition motifs of Ddi1 on substrates, particularly concerning its role in cleaving undegraded polyubiquitinated proteins.

Furthermore, the intricate relationship between Ddi1, Skn-1/Nrf1, and proteasome impairment, especially in the backdrop of resistance to treatments like Bortezomib, requires further investigation. The biological ramifications of Ddi1’s overexpression in response to DNA anomalies and its influence on cell cycle progression also present intriguing research prospects. Lastly, Ddi1’s potential role in proteostasis, given its capability to act on extensive polyubiquitinated chains and its synergistic role with the proteasome, is another area ripe for exploration.

Studies have demonstrated that the HIV protease inhibitor, nelfinavir, is effective against *Echinococcus multilocularis* larvae, targeting the Ddi1 protein (EmuDdi1) and against *Leishmania* [54]. Nelfinavir inhibits the proteolytic activity of recombinant EmuDdi1, blocking pathways associated with EmuDdi1-mediated protein export. This indicates a significant role of the RVP domain in interacting with HIV inhibitors [74].

The intricate involvement of Ddi1 in protein quality control pathways positions it as a promising target for therapeutic intervention, particularly in diseases characterized by protein misfolding or aggregation, such as neurodegenerative disorders and certain cancers. By modulating the activity of Ddi1, it may be possible to enhance cellular proteostasis and ameliorate pathological conditions. Demonstrating similar proteolytic activity to the proteasome suggests a potential avenue for the development of pharmaceutical agents akin to those employed in the proteasome inhibition. However, the development of drugs targeting Ddi1 is not devoid of challenges. One major hurdle lies in achieving selectivity, as Ddi1 shares structural similarities with other ubiquitin receptors, necessitating the design of compounds that specifically target Ddi1 without interfering with related proteins. The utilization of pharmacological compounds analogous to drugs employed in HIV treatment offers promise for the generation of selective inhibitors.

New research trajectories promise to shed light on the multifaceted roles of Ddi1 and its domains, potentially unlocking new therapeutic applications and deepening our understanding of cellular processes.

## Data Availability

Not applicable.
